# A Neuroimaging Perspective on Familial Alzheimer's Functional Data through Bayesian Eyes

**DOI:** 10.1002/alz.085184

**Published:** 2025-01-09

**Authors:** Alice Grazia, Martin Dyrba, Pomara Nunzio, Anna G.M. Temp, Michel J. Grothe, Stefan Teipel

**Affiliations:** ^1^ Rostock University Medical Centre, Rostock Germany; ^2^ Deutsches Zentrum für Neurodegenerative Erkrankungen (DZNE), Rostock‐Greifswald Standort, Rostock Germany; ^3^ Deutsches Zentrum für Neurodegenerative Erkrankungen e. V. (DZNE), Rostock/Greifswald, Rostock Germany; ^4^ NYU School of Medicine, New York, NY USA; ^5^ Nathan Kline Institute, Orangeburg, NY USA; ^6^ BG Klinikum Hamburg GmbH, Hamburg Germany; ^7^ Reina Sofia Alzheimer Center, CIEN Foundation, ISCIII, Madrid, Madrid Spain; ^8^ Deutsches Zentrum für Neurodegenerative Erkrankungen e. V. (DZNE), site Rostock / Greifswald, Rostock Germany; ^9^ Clinic of Psychosomatic Medicine and Psychotherapy, University Medical Center Rostock, Rostock Germany

## Abstract

**Background:**

Familial Alzheimer's disease research necessitates innovative methodologies to disentangle the intricate relationships between genetic factors and neuroimaging measures. Traditional frequentist approaches, often hampered by small sample sizes in this population and challenges in incorporating prior knowledge transparently, may limit the robustness of findings.

**Methods:**

We analyzed neuroimaging data of preclinical PSNE1 single mutation carriers, utilizing the software JASP to test effects of carrier status on measures of basal forebrain functional connectivity using both frequentist and Bayesian approach. Bayesian Analysis of Covariance was first implemented to test the hypothesis of a basal forebrain functional connectivity reduction in mutation carriers compared to non‐mutation carriers. To get more precise information, Informative priors, derived from the existing literature on preclinical sporadic Alzheimer's disease, were integrated into post‐hoc Independent samples t‐tests. Student t‐tests were utilized for parameter estimation, using an Informed Cauchy specification of prior's location (‐0.7) and scale (0.707) allowing the derivation of posterior distributions.

**Results:**

Our findings demonstrated that preclinical mutation carriers exhibited no significant alterations in basal forebrain functional connectivity compared to non‐carriers. However, the Bayesian analysis revealed distinct advantages over the traditional frequentist approach (anterior basal forebrain: t=‐1.126 (213), p= 0.131, Cohen's d = ‐0.165, 95 % CI for Cohen's d: [‐Inf., 0.077]; posterior basal forebrain: t=‐1.337 (213), p= 0.091, Cohen's d = ‐0.196, 95 % CI for Cohen's d: [‐Inf., 0.046]. Incorporating informative priors significantly improved the precision of parameter estimates, allowing us to reject our hypothesis with a relatively high degree of confidence, from anecdotal (anterior basal forebrain: BF_10_ = 1.167, δ = ‐ 0.319, 95 % CI: [‐0.599, ‐0.061]) to strong evidence (posterior basal forebrain: BF_10_ = 0.033, δ = ‐0.067, 95 % CI: [‐0.260, ‐0.003]).

**Discussion:**

Our results underscore the importance of adopting Bayesian frameworks in familial Alzheimer's disease research. The explicit integration of prior knowledge, as facilitated by Bayesian analysis, enhances the reliability of conclusions drawn from neuroimaging data. These methodological advantages are crucial in advancing the field and encouraging replication efforts.

